# Morphological Effect of Pd Catalyst on Ethanol Electro-Oxidation Reaction

**DOI:** 10.3390/ma5091686

**Published:** 2012-09-19

**Authors:** Raúl Carrera Cerritos, Minerva Guerra-Balcázar, Rosalba Fuentes Ramírez, Janet Ledesma-García, Luis Gerardo Arriaga

**Affiliations:** 1 Center for Research and Technological Development in Electrochemistry, Parque Tecnológico Querétaro s/n, Sanfandila, Pedro Escobedo, Qro., C.P. 76703, México; E-Mail: maracac21@hotmail.com; 2 Chemical Engineering Department, University of Guanajuato, Natural Sciences Division, Noria Alta s/n, Col. Noria Alta, Guanajuato, Gto., C.P. 36050, México; E-Mail: rfuentes@ugto.mx; 3 Research and Graduate Division, Engineering Faculty, Autonomous University of Queretaro, Cerro de las Campanas, Querétaro, Qro., C.P. 76010, México; E-Mails: minbalca@yahoo.com.mx (M.G.B.); janet.ledesma@uaq.mx (J.L.G.)

**Keywords:** Pd nanoparticles, Pd nanobars, Pd nanorods, ethanol electro-oxidation

## Abstract

In the present study, three different structures with preferentially exposed crystal faces were supported on commercial carbon black by the polyol method (nanoparticles (NP/C), nanobars (NB/C) and nanorods (NR/C)). The electrocatalysts were characterized by XRD, TEM, TGA and cyclic voltammetry at three different ethanol concentrations. Considerable differences were found in terms of catalytic electroactivity. At all ethanol concentrations, the trend observed for the ethanol oxidation peak potential was preserved as follows: NB/C < NP/C< NR/C < commercial Pd/C. This result indicates that, from a thermodynamics point of view, the NB/C catalyst enclosed by Pd(100) facets presented the highest activity with respect to ethanol electro-oxidation among all of the catalysts studied.

## 1. Introduction 

World energy production is dominated by fossil fuels. Nevertheless, growing concerns regarding the depletion of petroleum-based energy resources, environmental contamination, and the increase in fuel prices due to limited oil reserves have motivated intensive research to substitute renewable energy sources for fossil fuels [1]. One possible way of obtaining energy from renewable sources involves the fermentation of organic matter to produce bioethanol, which is a low-toxicity liquid that features high power density (8 KWh/kg) and is easy to transport [2]. It has been suggested that instead of using bioethanol in internal combustion engines it could be used to generate hydrogen through steam-reforming reactions for more efficient fuel-cell applications [3,4,5]. An alternative way of producing energy from ethanol has recently been proposed. This consists of using a Direct Ethanol Fuel Cell (DEFC) to perform the Ethanol Electro-oxidation Reaction (EOR) directly over an anodic electrocatalyst. The problem with using ethanol as a fuel is that the EOR is difficult to induce and occurs at slower rates compared to modern electrocatalysts. This typically leads to the incomplete oxidation of ethanol, which reduces the efficiency of fuel cells and can lead to the creation of by-products or electrode deactivation [6]. However, studies on EOR have been performed in recent years, and it is expected that substantial improvements in the EOR can still be made [7]. 

Although Pd is nearly inert to the EOR in acid media, it has demonstrated competitive EOR activity and a slightly better ability to break the C-C bond of ethanol in high pH media compared to Pt-based catalysts. This opens the opportunity of using Pd instead of Pt, thus reducing the cost of fuel cells. In addition, the abundance of Pd in the Earth’s crust is 200 times higher than that of Pt [8,9].

Recently, it has been shown that the electrocatalytic activity and selectivity of a catalyst can be greatly enhanced by using nanocrystals enclosed by specific crystal facets that are intrinsically more active with respect to a particular reaction. For example, polygonal Pd catalysts with dominant (111) facets show excellent thermal stability and catalytic activity with respect to formic acid electro-oxidation compared with common Pd/C catalysts [10]. In addition, a strong morphological dependence of the catalytic activity of Pd toward the oxygen reduction reaction (ORR) has been reported. Upon changing the morphology of Pd catalysts from that of nanoparticles to that of nanorods with preferential (110) facets by electro-deposition, the specific activity increases 10-fold and becomes comparable to that of Pt under the operating potentials of fuel cells [11]. Furthermore, Pt nanocube catalysts with dominant (100) faces show a lower onset potential and higher current density during methanol and ethanol electro-oxidation than polycrystalline Pt catalyst in acid media [12]. 

The EOR over Pd surfaces is a typical multistep and multiselective catalytic process with a complex network of elementary steps. However, the structure of Pd catalysts can greatly affect the EOR due to the various atomistic properties and adsorption behaviors of different Pd crystal facets. In this respect, the adsorption behavior and oxidation mechanisms of ethanol over Pd surfaces, including (111), (100) and (110), have been studied by Density Functional Theory (DFT) in recent years. The results show, for the first time, that the activity and selectivity of ethanol oxidation on Pd are highly structure-sensitive and prove that Pd(100) is the best surface for the dissociation of an ethanol molecule, with a rather low energy barrier [13]. Nevertheless, unlike those performed for Pt-based catalysts, theoretical and experimental structure-sensitivity studies related to the EOR over Pd surfaces is scarce.

In this study, preferential Pd structures were synthesized, characterized and supported on commercial carbon black (C). The polyol synthesis method was used to prepare three different structures, nanoparticles (NP/C), nanobars (NB/C) and nanorods (NR/C), which were used as anode electrocatalysts for the ethanol oxidation reaction. The catalysts were physicochemically characterized by Electron Microscopy Transmission (TEM), Thermo-Gravimetric Analysis (TGA) and X-Ray Diffraction (XRD). The nanostructures were electrochemically evaluated for EOR in alkaline medium using Cyclic Voltammetry (CV) at three ethanol concentrations (0.1, 1 and 3 M). 

## 2. Materials and Methods

### 2.1. Synthesis of Catalysts 

The catalyst was synthesized using a previously reported method, which was slightly modified [14]. In the case of the NP/C catalysts, 5 mL of ethylene glycol (EG, J.T. Baker, Austin, TX, USA, 99.9%) was placed in a 25 mL three-neck flask equipped with a reflux condenser and a Teflon-coated magnetic stirring bar. The flask was heated in static air under magnetic stirring at 373 K. Meanwhile, 0.0486 g of Na_2_PdCl_4_ (Sigma-Aldrich, St. Louis, MO, USA, 98%) was dissolved in 3 mL of deionized water, and 0.0916 g of polyvinylpyrrolidone (PVP, Aldrich, St. Louis, MO, USA, Mw = 55,000) was dissolved in 3 mL of EG at room temperature. These two solutions were then injected simultaneously into the flask using a two syringe pumps (Cole Palmer Instruments Company, Vernon Hills, IL, USA) at a rate of 45 mL h^−1^. The reaction mixture was heated at 373 K while 0.037 g of carbon black previously heat treated at 550 K for 3 h was added during the reduction process. After 1 h, the reaction was cooled to room temperature before the product was collected by filtration and washed with deionized water to remove most of the EG and excess PVP. Finally, the product was dried at 333 K for 12 h. A similar process was employed to produce the NR/C catalyst, except that 0.6 g KBr (J.T. Baker, Austin, TX, USA, 99%) was added in the Pd precursor solution to promote unidirectional growth. For the NB/C catalyst, a procedure similar to that used to produce the NR/C catalyst was followed, except that diethylene glycol (DEG) (Aldrich, St. Louis, MO, USA, 99%) instead of EG was used to perform the reaction [14].

### 2.2. Materials Characterization 

The crystallinity of the catalysts was investigated using a Bruker D8 Advance, X-ray diffractometer, operated using Cu-Kα radiation at 30 kV and 30 mA over a 2θ range of 20–130 with a step size of 0.009151°. TEM analyses were conducted on a Jeol JEM-100S apparatus operated at 60 kV to observe the morphology and crystal size of the catalysts. The Pd load deposition was measured by TG analysis of the prepared catalyst, which was carried out using a thermogravimetric balance (TA Instruments, Q500). Thermogravimetric analysis was performed from room temperature to 1073 K at a heating rate of 10 K min^−1^ under an air flow rate of 60 cm^3^ min^−1^. 

### 2.3. Electrochemical Measurements

All electrochemical measurements were conducted on a potentiostat/galvanostat BASi-Epsilon in a three-electrode cell at room temperature. An Hg/HgO in 1 M KOH and a platinum foil electrode were used as the reference electrode and counter electrode, respectively. The working electrode was prepared using a glassy carbon (GC) disc measuring 3 mm in diameter, which was previously polished with alumina powder (0.05 μm), sonicated for 10 min and washed with deionized water. During the electrochemical measurements, a mixture containing 1.0 mg of electrocatalyst and 73 μL of isopropyl alcohol (Baker, 99.9%) was pretreated for 20 min under ultrasonication. Then, 7 μL of Nafion^®^ solution (5% isopropyl alcohol, Electrochem) was added to the mixture and sonicated for 20 min again to obtain a well-dispersed ink. A 2.2 μL portion of the catalyst ink was then transferred onto the surface of the GC electrode and dried in air to obtain a catalyst thin film. Cyclic voltammetry (CV) was performed in aqueous solutions of 1 M KOH in both the absence and presence of ethanol solutions of various concentrations from −1 to 0.4 V (*vs*. NHE) at 0.05 V s^−1^.

## 3. Results and Discussion

### 3.1. Materials Characterization

#### 3.1.1. XRD Analysis

**Figure 1:**
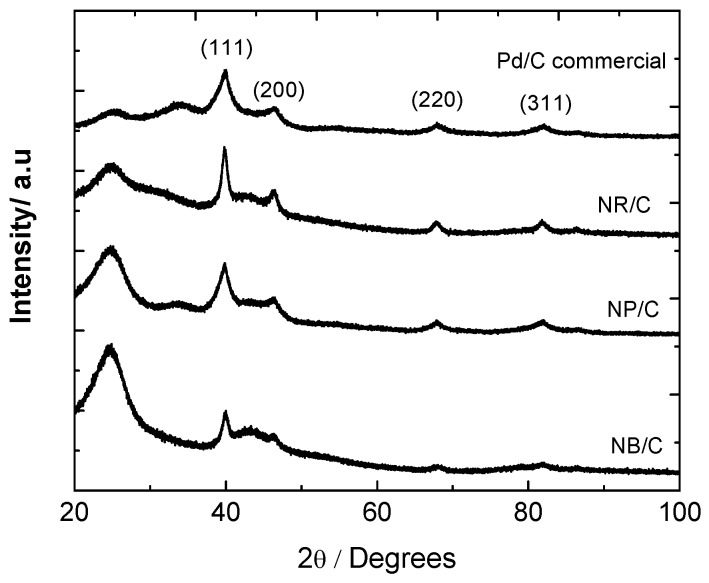
XRD patterns of commercial Pd, NR/C, NB/C and NP/C catalysts.

The XRD diffraction patterns of NR/C, NB/C and NP/C are shown in Figure 1. The XRD reflections showed face-centered cubic lattice structures with diffraction peaks at Bragg angles of 2θ = 40.119, 46.659, 68.121, 82.1, 86.619 and 119.334°, corresponding to the (111), (200), (220), (311), (222) and (331) planes of the Pd metallic phase (JCPDS 46-1043, Figure 1a), respectively. In addition, a reflection at 2θ~25° was observed, which was assigned to the (002) plane of the carbon black, and a broad peak centered at 2θ~35° was observed on the commercial Pd/C and NP/C catalysts, which was likely due to the main reflection of PdO∙H_2_O (JCPDS card 09-0254). The latter peak indicates the incomplete reduction of these catalysts or surface re-oxidation after the purification step. The average crystal size was calculated based on the broadening of the (111) diffraction peak according to the Scherrer Equation [15]:

$$d=\frac{0.9\lambda }{B_{2\theta }\cos\theta_{\max}}$$

Where λ is the wavelength of the incident X-ray (0.1542 nm for Cu-Kα radiation); θ is the angle of the maximum peak; and B_2θ_ is the width of the half-height peak [15]. The average crystal sizes calculated for the commercial Pd/C, NR/C, NB/C and NP/C catalysts were 4.9, 10.2, 9 and 6.4 nm, respectively. The (111) to (200) intensity ratios calculated for the commercial Pd/C, NR/C, NB/C and NP/C catalysts were 2.82, 2.77, 2.71 and 2.94, respectively. This suggests that the NB/C catalysts showed the most dominant (200) plane of all the catalysts that were studied. Furthermore, the NP/C catalyst shows a highly dominant (111) plane. Similar results were reported for polyhedral Pd/C catalysts synthesized by the polyol process [10].

#### 3.1.2. Thermogravimetric Analysis.

**Figure 2:**
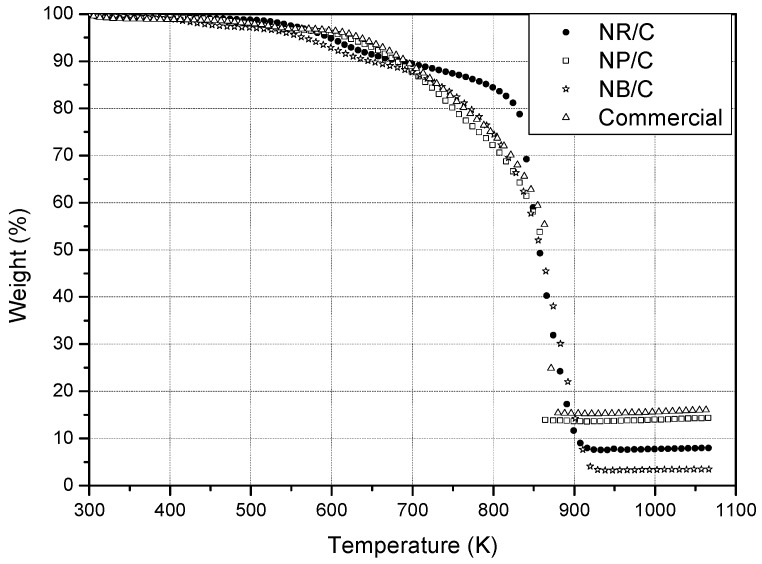
TGA curves of commercial Pd/C, NR/C, NB/C, and NP/C catalysts.

The TGA curves of the catalysts are presented in Figure 2. The behavior observed was similar for all of the prepared catalysts and similar to that reported for other Pd/C catalysts [8,16]. The TGA curves show three distinct regions: the slight loss in weight at temperatures below 600 K may have been caused by the evaporation of adsorbed water and the residual organic solvent. The second region consists of a significant decrease in the weight fraction at temperatures in the range of 650–900 K, which is mainly associated with the decomposition of complex organics; this indicates that the carbon reacted with O_2_ to form CO_2_ at temperatures above 850 K. The stable weight fractions corresponding to the real Pd loading on the carbon were found to be 15.4, 13.7, 7.5 and 3.3 for the commercial Pd/C, NP/C, NR/C and NB/C, respectively. The low Pd loading in the NR/C may be related to the presence of Br^-^ ions because they can suppress the deposition of Pd atoms by protecting the surface through adsorption [17]. In addition, the lowest Pd load found in the NB/C catalyst was associated not only with the ion etching of Br^−^ but also through the weaker reducing power of the DEG, which employed in the present work to slow the reduction rate required to achieve NB formation [14]. 

#### 3.1.3. TEM Observations

**Figure 3:**
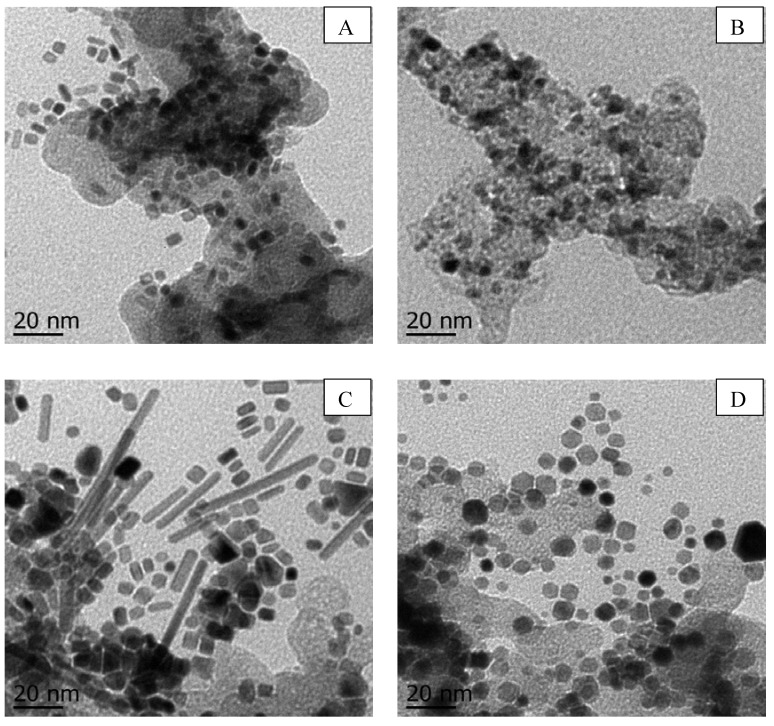
TEM images of (**A**) NB/C; (**B**) commercial Pd/C; (**C**) NR/C; and (**D**) NP/C catalysts.

The morphology of the Pd catalysts supported on carbon black is shown in Figure 3. As shown in Figure 3a, most of the nanoparticles consisted of Pd nanobars with an average particle size of approximately 4 (width) × 4 nm (large) to 4 (width) × 10 nm (large). This implies that compared to the previous synthesis of unsupported nanobars, where the particle size was ~ 8 × 8 nm to 8 × 10 nm, similarly shaped and thinner Pd nanobars were successfully prepared and supported on carbon black [14]. The average particle sizes of the commercial Pd/C and NP/C catalysts (Figure 3b,d respectively) were 6 and 5 nm, respectively; these results are in good agreement with the results of the XRD analysis. Moreover, the high-magnification TEM images indicate that the commercial Pd/C catalyst is composed of irregularly shaped Pd nanoparticles, while the NP/C catalyst is composed of polygonal Pd nanostructures. This result was expected because the NP/C catalysts were produced by modifying the synthesis method reported by Xiong *et al*. to produce unsupported Pd nanoparticles [14]. This suggests that carbon black did not participate in the formation of the nanostructures. The NR/C catalyst (Figure 3c) featured nanorods of various lengths ranging from ~5–80 nm, with a measured diameter of 5 nm. These nanorods are longer than those reported by Xiong *et al*. [14], most likely due to the higher reaction temperature employed in the present study (388 K versus 373 K). 

### 3.2. Electrochemical Characterization

#### 3.2.1. Cyclic Voltammetry in Absence of Ethanol

**Figure 4:**
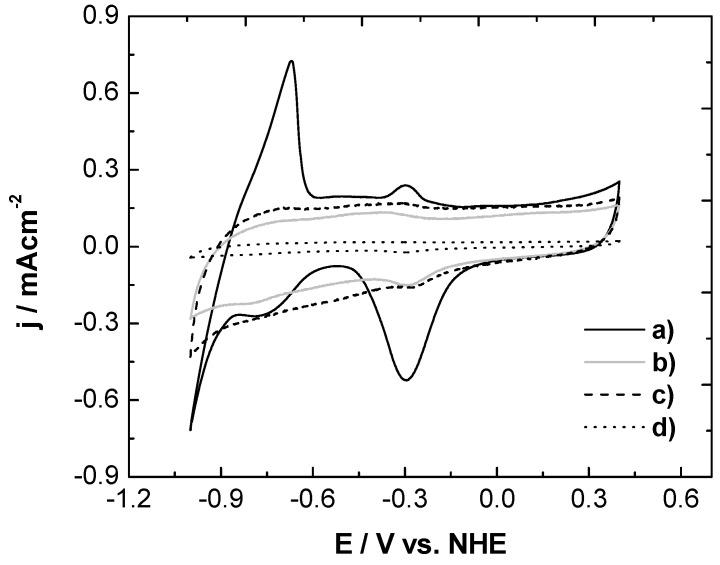
Cyclic voltammograms of (**a**) commercial Pd/C; (**b**) NR/C; (**c**) NB/C; and (**d**) NP/C catalysts in 1 M KOH at room temperature.

Figure 4 shows the voltammetric curves of the NP/C, NB/C and NR/C and Pd commercial catalysts in 1 M KOH solution at room temperature. The current was normalized by the specific surface area (ESA) and the metal loading of each catalyst. Three potential peaks were observed in the anodic scan, which correspond to different electrochemical processes occurring over the Pd surface. The peaks that appear at potentials lower than –0.75 V are associated with the oxidation of absorbed and desorbed hydrogen, according to Equation (1) [18]. It was noted that the NB/C and NR/C catalysts presented lower hydrogen absorption than commercial Pd/C catalysts:

Pd-H_abs/ads_ + OH^−^ → Pd + H_2_O + e^−^ (1)

The second peak occurs at potentials of ~ −0.3 V and is related to the adsorption of hydroxyl groups, according to Equation (2) [19]. These peaks in the NR/C and NB/C catalysts shifted towards more negative potentials compared to the peaks of the commercial Pd/C catalyst. This implies that the synthesized NB/C and NR/C presented a higher affinity for hydroxyl group adsorption compared to the commercial Pd/C catalysts. This observation will be further discussed within the context of the half-cell experiments:

Pd + OH^−^ ↔ Pd-OH_ads_ + e^−^ (2)

The third peak occurs at potentials above −0.2 V. Although this signal is associated with a process that has not been fully explained, it is generally accepted that OH^-^ ions are first chemisorbed during the initial stage of oxide formation and then are transformed into higher-valence oxides at higher potentials (Equations 3,4). On the other hand, a peak centered at ~ −0.3 V was observed in the cathodic scan, which is associated with the reduction of Pd (II), according to Equation (5):

Pd-OH_ads_ + OH^−^ ↔ Pd-O + H_2_O + e^−^ (3)

Pd-OH_ads_ + Pd-OH_ads_ ↔ Pd-O + H_2_O (4)

Pd-O + H_2_O + 2e^−^ ↔ Pd + 2OH^−^ (5)

#### 3.2.2. Cyclic Voltammetry in Presence of Ethanol

**Figure 5:**
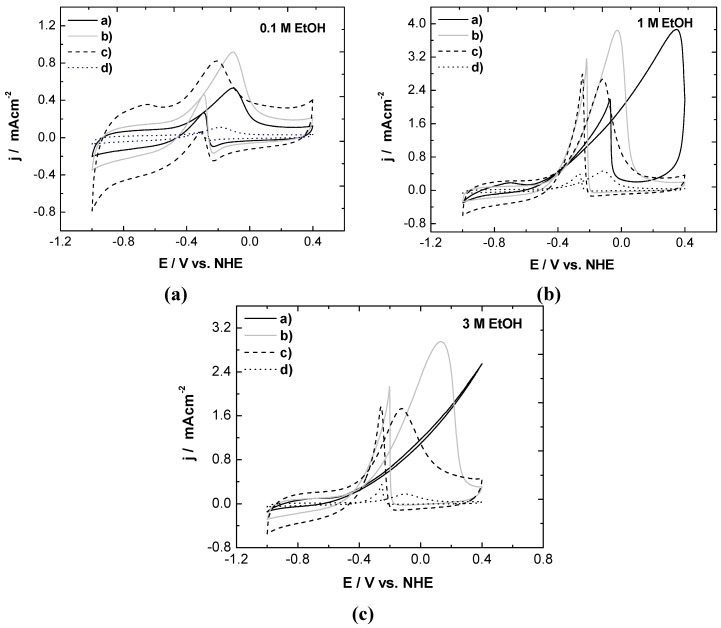
Cyclic voltammograms of Pd catalysts in 1 M KOH^+^: (**a**) 0.1; (**b**) 1; and (**c**) 3 M ethanol at room temperature.

Figure 5 shows the ethanol electro-oxidation of the electrocatalysts with different morphologies in the 1 M KOH. The previously proposed oxidation sequence for ethanol oxidation in alkaline media sets the ethanol adsorption as the first step followed by further dissociation, according to Equations (6,7) [18]:

Pd + CH_3_CH_2_OH ↔ Pd-(CH_3_CH_2_OH)_ads_ (6)

Pd-(CH_3_CH_2_OH)_ads_ → Pd-(CH_3_CO)_ads_ + 3H_2_O +3e^-^ (7)

**Figure 6:**
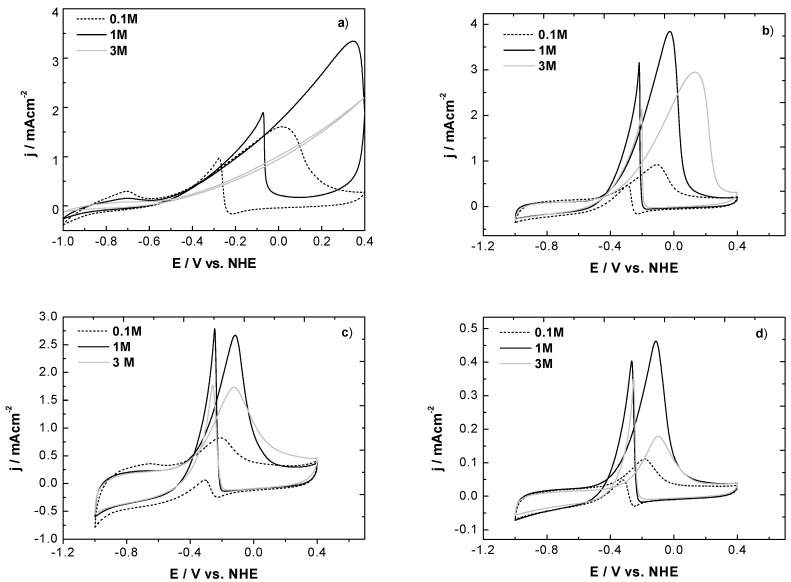
Cyclic voltammograms of Pd catalysts in 1 M KOH at room temperature in three ethanol concentrations: (**a**) commercial Pd/C; (**b**) NR/C; (**c**) NB/C; and (**d**) NP/C.

Thus, the suppression of the hydrogen absorption-desorption signal (Equation 1) was observed in the presence of ethanol, which became more evident in the commercial Pd/C and NB/C catalysts as the ethanol concentration increased; this was attributed to the dominant dissociative adsorption of ethanol in the low-potential region at high alcohol concentrations (Figure 6).

All voltammograms were characterized by two well-defined current peaks: one in the anodic scan and the other in the cathodic scan. In the anodic scan, the oxidation peak (i_f_) was related to the oxidation of freshly chemisorbed species derived from ethanol adsorption. The oxidation peak in the reverse scan (i_r_) is associated with the removal of carbonaceous species that are not completely oxidized in the positive scan, rather than the oxidation of freshly chemisorbed species [20]. 

The voltammograms shown in Figure 5 were analyzed in terms of the peak potentials E_f_ shown in Table 1. For example, the E_f_ values measured from the voltammograms (Figure 5a) at 0.1 M ethanol were 226, 102 and 27 mV more positive than those of the NB/C catalysts for the commercial Pd/C, NR/C and NP/C catalyst, respectively. Moreover, the trend observed for E_f_ was preserved in all ethanol concentrations as follows: NB/C < NP/C < NR/C < commercial Pd/C. At all ethanol concentrations, the NR/C catalyst presented the highest peak current density, followed by the NB/C and NP/C catalysts. The E_f_ obtained for the NB/C catalysts shifted towards more negative potentials relative to the values of the other catalysts, independently of the ethanol concentration.

**Table 1:** E_f_ of Commercial Pd/C, NR/C, NB/C, and NP/C catalysts obtained in 1 M KOH solution at room temperature*.*

Ethanol concentration	E_f_/V			
	Commercial	NB/C	NR/C	NP/C
0.1 M	0.016	−0.210	−0.108	−0.183
1 M	0.347	−0.117	−0.026	−0.113
3 M	-	−0.122	0.132	−0.099

Density Functional Theory studies reported, for the first time, that the activity and selectivity of ethanol oxidation on palladium are highly structure-sensitive and prove that Pd(100) is the best surface for the dissociation of an ethanol molecule due to the strong ethanol-Pd(100) interaction, which leads to the lowest energy barrier relative to those of the Pd(111) and Pd(110) surfaces [13]. From the point of view of preferential orientations, the results shows that the NB/C catalyst presented the highest activity of all of the catalysts studied, which is consistent with the previously mentioned DFT calculus.

The effect of ethanol concentration on the EOR for the Pd catalysts was also analyzed in the present study and is illustrated in Figure 6. Higher current densities were observed when the ethanol concentration was increased from 0.1 to 1 M. However, the current density was reduced at an ethanol concentration of 3 M. The calculated reductions in the current density were 42, 26, and 65% for the NB/C, NR/C and NP/C, respectively. On the other hand, the E_p_ shifted towards more positive potentials as the ethanol concentration increased. This trend was more significant in the commercial Pd/C and NR/C catalysts. In fact, the oxidation peak was absent in the window employed at an ethanol concentration of 3 M for the commercial Pd catalysts. Furthermore, the ethanol oxidation peak remained at nearly the same potential for the NB/C catalyst (see Table 1 and Figure 6d). It has been previously suggested that the rate-limiting step of ethanol electro-oxidation involves the reaction of ethoxy species with OH^−^ through Equation (8). Moreover, OH^-^ adsorption is assumed to be independent of ethanol concentration [18]:

Pd-(CH_3_CO)_ads_ + Pd-OH_ads_ → Pd-CH_3_COOH + Pd (8)

On the other hand, the surface area covered by ethoxy species can be gradually increased as the ethanol concentration increases (see Equation 7) leading to higher current densities. At high ethanol concentration (3 M), the suppression of hydrogen absorption-desorption processes indicates that ethanol adsorption is enhanced, leading to a greater amount of ethoxy species over the Pd surface, whereas the adsorption of hydroxyl groups is largely blocked by CH_3_CO species as the potential increases. As indicated by Equation (8), the insufficient coverage of OH^-^ ions will lead to a decrease in the peak current, as the peak current is only achieved with an intermediate coverage of the ethoxy and hydroxyl adsorbates.

## 4. Conclusions

Different Pd structures with preferentially exposed crystal faces were synthesized and supported on commercial carbon black by the polyol method. Although a lower Pd load (determined by TGA) was achieved in the catalysts prepared in the present study, considerable differences were found in terms of catalytic electroactivity. At all ethanol concentrations, the NR/C catalyst presented the highest peak current density, followed by the NB/C and NP/C. Moreover, the trend observed for the ethanol oxidation peak potential was preserved at all ethanol concentrations as follows: NB/C < NP/C < NR/C. This result is supported by previously reported DFT results and indicates that, from a thermodynamic point of view, the NB/C catalyst enclosed by Pd(100) facets (orientations inferred from XRD and TEM analysis) presented the highest activity towards ethanol electro-oxidation compared to all of the studied catalysts. 
